# Prevalence of and factors associated with acute diarrhea among children under five in rural areas in Ethiopia with and without implementation of community-led total sanitation and hygiene

**DOI:** 10.1186/s12887-022-03202-8

**Published:** 2022-03-21

**Authors:** Gezahegn Mernie, Helmut Kloos, Metadel Adane

**Affiliations:** 1 Menz Gera Midir District Health Office, Environmental Health and Hygiene Unit, North Shoa Zone, Amhara Region Ethiopia; 2 Department of Epidemiology and Biostatistics, University of California, San Francisco, USA; 3 Department of Environmental Health, College of Medicine and Health Sciences, Wollo University, Dessie, Ethiopia

**Keywords:** Acute diarrhea, Children under five, Community-Led Total Sanitation and Hygiene, Rural areas, Ethiopia

## Abstract

**Background:**

Since Ethiopia has been implemented the Community-Led Total Sanitation and Hygiene (CLTSH) approach to control incidence of diarrhea, few studies have compared the prevalence of diarrhea and associated factors in rural areas that have implemented this approach with those that have not implemented it, and none have examined it in the district of Menz Gera Midir in the Amhara Region of Ethiopia. This study addressed this gap.

**Method:**

A community-based comparative cross-sectional study was conducted among 224 children under five in three randomly selected rural *kebeles* (the smallest administrative units in Ethiopia) where CLTSH had been implemented and 448 similar children in three other randomly selected rural *kebeles* where CLTSH had not been implemented during February and March, 2020. Data were collected using a structured questionnaire and an on-the-spot observational checklist. Data were analyzed using three different binary logistic regression models with 95% confidence interval (CI): the first model (Model I) was used for CLTSH-implementing *kebeles*, the second model (Model II) for non-CLTSH-implementing *kebeles*, and the third model (Model III) for pooled analysis of CLTSH-implementing and non-implementing *kebeles*. To control confounders, each multivariable logistic regression model was built by retained variables with *p* < 0.25 from the bi-variable logistic regression analysis. From the adjusted multivariable analysis of each model, variables with *p*-values < 0.05 were declared factors significantly associated with acute diarrhea.

**Results:**

The prevalence of acute diarrhea among children under five from households in *kebeles* that had implemented CLTSH was 10.6% (95% CI:6.6–14.7%) and among those that had not implemented CLTSH 18.3% (95%CI:14.8–22.2%). In CLTSH-implementing areas, use of only water to wash hands (AOR: 3.28; 95% CI:1.13–9.58) and having a mother/caregiver who did not wash their hands at critical times (AOR: 3.02; 95% CI:1.12–8.12) were factors significantly associated with acute diarrhea. In non-CLTSH-implementing areas, unimproved water source (adjusted odds ratio [AOR]: 2.81; 95% CI:1.65–4.78), unsafe disposal of child feces (AOR: 2.10; 95% CI:1.13–3.89), improper solid waste disposal (AOR: 1.95; 95% CI:1.12–3.38), and untreated drinking water (AOR: 2.33; 95% CI:1.21–4.49) were factors significantly associated with acute diarrhea. From the pooled analysis, not washing hands at critical times (AOR: 2.54; 95% CI:1.59–4.06), unsafe disposal of child feces (AOR: 2.20; 95% CI:1.34–3.60) and unimproved water source (AOR: 2.56; 95% CI:1.62–4.05) were factors significantly associated with the occurrence of acute diarrhea while implementation of CLTSH was a preventive factor (AOR: 0.24; 95%: 0.20–0.60) for the occurrence of acute diarrhea.

**Conclusion:**

The prevalence of acute diarrhea among under-five children in Menz Gera Midir District was lower in *kebeles* where CLTSH had been implemented than in *kebeles* where CLTSH had not been implemented. Therefore, we recommend that governmental and non-governmental sectors increase implementation of CLTSH programs, including improving handwashing at critical times, promoting safe disposal of child feces and enhancing the availability of improved water sources.

**Supplementary Information:**

The online version contains supplementary material available at 10.1186/s12887-022-03202-8.

## Introduction

Diarrhea is defined as three or more loose or watery stools in a 24-h period [1]. It may be caused by a number of bacterial, viral, protozoan, or parasitic organisms. In developed and developing countries, rotavirus and *Escherichia coli* are the most common etiological agents of diarrheal disease. Diarrheal diseases are more common in communities with poor sanitation, poor hygiene practices, a lack of safe water for drinking, improper child feeding practices, and poor housing conditions [2].

Globally, diarrhea kills more children than AIDS, malaria, and measles combined [3]. Annually, 1.9 million children die from diarrheal diseases. About 78% of children who die from diarrhea live in Africa and Southeast Asia [4]. The major contributors to diarrheal disease are poor sanitation, lack of hygiene, and lack of safe drinking water [5].

In Africa’s sub-Saharan countries including Ethiopia, where hygiene and sanitation are poor, the incidence of diarrheal diseases is highest. Diarrhea is the leading cause of morbidity and mortality in children under five years of age in Africa. African children experience, on average, five episodes of diarrhea every year, and an estimated 800,000 die from diarrhea and dehydration [6]. About 80% of the rural population and 20% of the urban population of sub-Saharan Africa lack access to safe drinking water and sanitation [7].

In Ethiopia, childhood diarrheal disease is most common among 6- to 11-month-old children; the percentage of children under five years old who had diarrhea in a two week period decreased from 24% in 2000 to 18% in 2005 [8], 13% in 2011 [9], and 12% in 2016 [5]. Acute diarrhea is a common problem in Menz Gera Midir District, Amhara Region, Ethiopia. In its 2018/19 annual performance report, the district health office listed diarrhea as the leading cause of under-five morbidity. Out of 12,631 children under five, 2,023 (16.02%) sought treatment for diarrhea at a health institution [10].

One strategy for the prevention of diarrhea in Ethiopia is the implementation of Community-Led Total Sanitation (CLTS), now known as Community-Led Total Sanitation and Hygiene (CLTSH) [11]. The 2011 Ethiopian Hygiene and Sanitation Strategic Action Plan indicated that CLTSH had reached all nine regions of Ethiopia, but had not been implemented in some rural areas [12]. The CLTSH approach is one of the most cost-effective ways to improve water, sanitation, and hygiene, especially in low-income countries and rural settings, where it can mobilize and sensitize communities to discontinue open defecation [11, 13], and serve as an important tool for changing the collective behavior of communities [11].

Since Ethiopia has been using the CLTSH approach to control incidence of diarrhea, few studies have compared the prevalence of diarrhea and associated factors in rural areas that have implemented this approach with those that have not implemented it. Thus, this study compared the prevalence of acute diarrhea and associated factors among rural children under five living in *kebeles* that had implemented CLTSH with similar children in *kebeles* that had not implemented CLTSH within Menz Gera Midir District, North Shoa Zone, Amhara Region, Ethiopia.

## Method

### Study area description and study design

A comparative cross-sectional study was conducted during February and March 2020 in Menz Gera Midir District, one of the 27 districts in North Shoa Zone of Amhara Region, Ethiopia. Children under five years of age in 2020 in the district was 13,422 [10]. The district consists of four urban *kebeles* (the smallest administrative units in Ethiopia) in Mehal Meda Town and 20 rural *kebeles*. Mehal Meda Town is the capital of the Menz Gera Midir District, which is located about 284 km north of Addis Ababa. Of the 20 rural *kebeles* in Menz Gera Midir District, 11 (55%) had implemented a CLTSH program and 9 (45%) did not employ the CLTSH approach in 2018 [10].

### Source and study population

The source population was all children under five in all rural *kebeles* in Menz Gera Midir District, whereas the study population was the selected under-five children in three CLTSH implementing and three non-implementing rural *kebeles* in Menz Gera Midir District. Children with bloody diarrhea *or* persistent diarrhea were excluded from the study.

### Sample size determination and sampling techniques

The sample size was calculated by the double proportion formula with the assumptions that a two-week prevalence of diarrhea among children under five in CLTSH-implementing *kebeles* was 9.9% [14] and non-implementing *kebeles* 22.22% [15] taken from studies in Dangla District and Kersa District, respectively; 80% power, ratio between CLTSH-implementing and non-implementing *kebeles* taken as 1:2, design effect of 1.5 and 10% non-response. The final sample size was 672, of which 224 were in CLTSH-implementing *kebeles* and 448 in non-implementing *kebeles* 448.

A two-stage sampling technique was used to select the study participants. In the first stage, six *kebeles* (three in CLTSH-implemented and another three in non-implemented *kebeles*) were selected by simple random sampling using the lottery method. Then, the sampling frame was prepared for each *kebele* by using the households with at least one child under five years of age. Then, based on the total study population of the selected *kebeles*, a sample size of 224 households was proportionally allocated to the three selected *kebeles* where CLTSH had been implemented. Similarly, a sample size of 448 households was proportionally allocated to the three selected *kebeles* where CLTSH had not been implemented.

In the second stage, a systematic random sampling technique was used to select specific households for inclusion in the study. A lottery method was used based on the respective K-value (5 for implementing and 10 for non-implementing *kebeles*) to select the first household in each *kebele*. In households with more than one child under five, one child was chosen using the lottery method to estimate the prevalence of diarrhea in the study population. Households in which the study participants were not present during data collection were revisited the same day. If they were again not available, another visit was made the next day in order to minimize the non-response rate. If not available after the third visit, they were considered as non-respondents.

### Acute diarrhea measurement

The outcome variable of this study was acute diarrhea, denoted as yes (1) or no (0), where yes indicated the presence of acute diarrhea and no indicated the absence of acute diarrhea during the two weeks prior to the survey. Diarrhea among children under five in CLTSH-implementing *kebeles* and non-implementing *kebeles* was identified by asking the participants’ mothers/caregivers questions based on WHO-defined signs and symptoms of diarrhea [16] that had occurred during the previous two weeks. The WHO protocol [16] does not specify the recall period and the type of diarrhea. Because our study focused on acute diarrhea, we adopted a two-week recall period as specified in the World Gastroenterology Organization’s Global Guidelines for acute diarrhea surveys [17]. We excluded bloody and persistent diarrhea since bloody diarrhea is frequently caused by dysentery and persistent diarrhea lasts more than 14 days [16, 17].

### Operational definitions

Definitions of independent variables are available in Table 1.

**Table 1:** Operational definitions of some independent variables

Variables	Operational definitions
Community-Led Total Sanitation and Hygiene (CLTSH)	An approach to changing sanitation and hygiene behavior rather than making physical changes in the community [18]
Caregiver	Any person who provides care for the child other than the mother [19]
Unimproved water sources	Unprotected dug well, unprotected spring, or surface water (river, dam, lake, pond, or stream) from which water was fetched [20]
Handwashing at critical times	Handwashing with soap after visiting latrine, after cleaning the bottom of a child, before preparing food, before eating, and before feeding a child [21]
Proper refuse disposal	Disposal of refuse by burning, burying in a pit, storing in a container or at a designated site [21]
Safe child feces disposal	Disposal of child feces in a latrine

### Data collection and quality assurance

Data were collected using a pre-tested questionnaire and an on-the-spot observation checklist. The questionnaire was developed after a review of the published literature. To ensure the quality and consistency of the data, the questionnaire was prepared in English, translated to Amharic and then back to English. During data collection, the data collector administered the questionnaire orally to the study participants using the Amharic language. The questionnaire and observation checklist consisted of socioeconomic, environmental, and behavioral information. The questionnaire was objective and logically sequenced. Before the actual data collection, the questionnaire was pre-tested on a sample 5% the size of the study sample in one CLTSH-implementing *kebele* and one non-implementing *kebele* near the study area to validate the data collection tool. The results of the pre-test were used to ensure clarity of language and verify skip patterns of the questions.

The questionnaire was administered by six nurses and two supervisors who had been trained by the principal investigator for two days on the data collection tools and procedures, including the aim of the study, content of the questionnaire, and how to approach study subjects. Supervisors oversaw interviewers daily during the whole period of data collection and checked questionnaires for completeness and consistency. During administration of the survey, the collected data were checked daily by the principal investigator and supervisors for completeness, and houses providing incomplete data were revisited once to obtain additional data.

Inter-observer reliability was ensured by providing clear definitions of the dependent and independent variables and events to be recorded, by training the data collectors, and by providing feedback about discrepancies during daily supervision, as explained elsewhere [22]. We re-interviewed 5% of the study participants using a different interviewer to check reliability of the information entered by different interviewers.

In order to verify the accuracy of data entries, two generic data verification strategies were employed [23]. As the first step, a randomly selected 10% of the questionnaires were thoroughly checked and then as the second step, the data were exported to the Statistical Package for the Social Sciences (SPSS) version 25.0 for data cleaning. To identify missing values and assess overall distributions, descriptive statistics of frequency distributions were examined. Basic data quality assurance measures were taken according to the study by Keleb et al., including data cleaning using browsing of data tables after sorting, graphical exploration of distributions using box plots, histograms, and scatter plots, frequency distributions and cross tabulations, summary statistics and statistical outlier detection using sorting [24].

### Data analysis

During data analysis, for normally distributed continuous variables, mean and standard deviation (SD) (mean ± SD) were calculated for continuous variables, whereas descriptive statistics such as frequencies (*n*) and percentage (%) were calculated for categorical data. Using the outcome variable of presence of acute diarrhea, we estimated the prevalence of acute diarrhea among the participating children for both CLTSH-implementing and non-implementing *kebeles.*

Data were analyzed using three different binary logistic regression models: the first model (Model 1) was used only for CLTSH-implementing *kebeles*, the second model (Model II) for non-CLTSH-implementing *kebeles*, and the third model (Model III) for pooled analysis of CLTSH-implementing and non-implementing *kebeles’* data. For each model, bi-variable and multivariable analysis were estimated and variables with *p* < 0.25 in bi-variable logistic regression were retained into multivariable analysis of each model for CLTSH-implementing and non-implementing *kebeles*.

From the adjusted multivariable logistic regression analysis, variables with *p*-value < 0.05 and adjusted odds ratio (AOR) at 95% CI (confidence interval) were declared significantly associated with acute diarrhea. A multicollinearity test was performed to assess the existence of correlation between the independent variables using a cut-point of standard error of 2; it showed that there was no multicollinearity, with a maximum standard error of 1.68. The Hosmer–Lemeshow goodness-of-fit test [25] with *p*-value greater than 0.05 was used for each model, finding the *p*-value of Model 1, Model 2 and Model 3 was 0.885, 0.932 and 0.971, respectively.

## Results

### Socio-demographic characteristics of study participants

A total of 654 households, 218 (33.3%) in CLTSH-implementing *kebeles* and 436 (66.7%) in non-implementing *kebeles*, were interviewed. The response rate was 97.3%. Most of the respondents (*n* = 505, 77.2%) were biological mothers and 149 (22.8%) were caregivers. Two-thirds of the respondents (*n* = 430, 65.7%) were in the age group 26–40 years, and 574 (87.7%) were married. Most mothers/caregivers (*n* = 356, 54.4%) reported having attended primary school. All participants were Orthodox Christians. Children’s ages ranged from 6 to 59 months, with a mean age of 29.4 months with SD ± 14.9. The family size of two-thirds of the participants (*n* = 432, 66.1%) was smaller than five persons (Table 2).

**Table 2:** Socio-demographic characteristics of participants in both CLTSH-implementing and non-implementing *kebeles* in Menz Gera Midir District, North Shoa, Amhara Region, Ethiopia, February to March 2020

Variable	Category	CLTSH status	
		**Implemented**	**Not implemented**
		***n* (%)**	***n* (%)**
Relation of respondent to child	Caregiver	37(17.0)	112(25.7)
	Mother	181(83.0)	324(74.3)
Age of respondent (years)	18–25	43(19.7)	68(15.6)
	26–40	148(67.9)	282(64.7)
	> 40	27(12.4)	86(19.7)
Sex of respondent	Female	195(89.4)	367(84.5)
	Male	23(10.6)	69(15.8)
Marital status of respondent	Single	12(5.5)	18(4.1)
	Divorced	10(4.6)	27(6.2)
	Widowed	2(0.9)	11(2.5)
	Married	194(89.0)	380(87.2)
Educational status of mother/caregiver	Illiterate	36(16.5)	71(16.3)
	Read & write	9(4.1)	15(3.4)
	Primary (1–8)	104(47.7)	252(57.8)
	Secondary or above	69(31.7)	98(22.5)
Educational status of father	Illiterate	21(9.6)	50(11.5)
	Read & write	9(4.1)	6(1.4)
	Primary (1–8)	85(39.0)	181(41.5)
	Secondary or above	79(36.2)	143(32.8)
Occupation of mother/caregiver	Housewife	183(83.9)	376(86.2)
	Farmer	33(15.1)	58(13.3)
	Merchant	2(0.9)	2(0.5)
Sex of child	Female	120(55.0)	214(49.1)
	Male	98(45.0)	222(50.9)
Family size (persons)	> 5	71(32.6)	151(34.6)
	1–5	147(67.4)	285(65.4)
Age of child (months)	< 11	23(10.6)	48(11.0)
	12–23	44(20.2)	92(21.1)
	24–35	51(23.4)	114(26.1)
	36–47	50(22.9)	85(19.5)
	48–59	50(22.9)	97(22.2)
Number of children under five in household	Two or more	22(10.1)	73(16.7)
	One	196(89.9)	363(83.3)
Birth order of child	First	94(43.1)	116(26.6)
	Second	61(28.0)	155(35.6)
	Third or above	63(228.9)	165(37.8)
Monthly household income ($, USD)	< 15.0	169(77.6)	341(78.2)
	15.0–22.7	28(12.8)	68(15.6)
	> 22.7	21(9.6)	27(6.2)

### Environmental characteristics

One hundred forty-eight (67.9%) of the households in CLTSH-implementing *kebeles* and 295 (67.7%) households in non-CLTSH-implementing *kebeles* used improved water sources. A latrine was available to 191 participants (87.6%) in CLTSH-implementing and 306 (70.2%) in non-implementing *kebeles.* Nine (4.1%) of the households in CLTSH-implementing and 23 (5.3%) households in the non-CLTSH-implementing *kebeles* shared latrines with another household. One hundred twenty-two (56.0%) and 131 (30%) of households in CLTSH-implementing and non-implementing *kebeles*, respectively, had handwashing facilities near the toilet (Table 3).

**Table 3:** Environmental conditions of study participants in CLTSH-implementing and non-implementing *kebeles*, Menz Gera Midir District, North Shoa Zone, Amhara Region, Ethiopia, February and March 2020

Variable	**Category**	CLTSH	
		**CLTSH-implementing *kebeles***	**Non-CLTSH-implementing *kebeles***
		***n* (%)**	***n* (%)**
Source of drinking water	Unimproved	70(32.1)	141(32.3)
	Improved	148(67.9)	295(67.7)
Time walking to fetch water (minutes)	> 30	148(67.9)	337(77.3)
	≤ 30	70(67.9)	99(22.7)
Average daily water consumption per person (liters)	< 20	172(78.9)	354(81.2)
	≥ 20	46(21.1)	82(18.8)
Water supply interruption	Yes	10(4.6)	37(8.5)
	No	208(95.4)	399(91.5)
Latrine availability	No	27(12.4)	130(29.8)
	Yes	191(87.6)	306(70.2)
Ownership of latrine	Shared	9(4.1)	23(5.3)
	Private	182(83.4)	287(65.8)
Type of latrine	Traditional	81(37.2)	279(64.0)
	Improved	111(50.9)	31(7.1)
Latrine has seat cover	No	99(45.4)	169(38.8)
	Yes	92(42.2)	141(32.3)
Number of households sharing latrine	> 2 households	6(2.8)	14(3.2)
	2 households	3(1.4)	9(2.1)
Child feces disposal	Outside the latrine	60(27.5)	264(60.6)
	Inside the latrine	158(72.5)	172(39.4)
Frequency of latrine cleaning	Never	44(22.0)	78(17.9)
	Sometimes	86(39.4)	187(42.9)
	Daily	61(27.9)	45(10.3)
Distance of latrine from kitchen (meters)	≤ 6	48(22.0)	55(12.6)
	> 6	143(65.6)	255(58.5)
Handwashing facility near toilet	No	69(31.7)	179(41.1)
	Yes	122(56.0)	131(30.0)
Refuse disposal	Improper	56(25.7)	195(44.7)
	Proper	162(74.3)	241(55.3)
Wastewater disposal	Improper	79(36.2)	168(38.5)
	Proper	139(63.8)	268(61.5)
Livestock kept in the house	Yes	53(24.3)	86(19.7)
	No	165(75.7)	350(80.3)

Child feces were properly disposed of in latrines among 158 (72.5%) households in *kebeles* that had implemented CLTSH and 172 (39.4%) households in *kebeles* that had not*.* One hundred sixty-two (74.3%) households in CLTSH-implementing *kebeles* and 241 (55.3%) in non-CLTSH-implementing *kebeles* disposed of their solid waste properly. Seventy-nine (36.2%) households in CLTSH-implementing *kebeles* and 168 (38.5%) households in non-CLTSH-implementing *kebeles* disposed of liquid waste improperly (Table 3).

### Behavioral characteristics

One hundred eighty-two (83.5%) mothers/caregivers in CLTSH-implementing *kebeles* and 359 (82.3%) in the non-implementing *kebeles* started supplementary feeding of infants at the age of six months. Two hundred seventeen (99.5%) children in the CLTSH-implementing *kebeles* and 427 (97.9%) in non-implementing *kebeles* had been vaccinated for rotavirus. More than half of the households in both the CLTSH-implementing (*n* = 125, 57.3%) and non-implementing (*n* = 303, 69.5%) *kebeles* did not treat drinking water at home. Sixty-three (28.9%) and 47 (10.8%) households in the implementing and non-implementing *kebeles*, respectively, treated drinking water by boiling. One hundred thirteen (51.8%) households in CLTSH-implementing and 242 (55.5%) households in non-implementing *kebeles* washed their hands at all the critical times, and 98 (45.0%) and 237 (54.4%) households in CLTSH-implementing *kebeles* and non-implementing *kebeles*, respectively, used water and soap for handwashing (Table 4).

**Table 4:** Behavioral factors of study participants associated with acute diarrhea, Menz Gera Midir District, North Shoa Zone, Amhara Region, Ethiopia, February and March 2020

Variable	Category	CLTSH	
		**CLTSH-implemented**	**Non-CLTSH-implemented**
		***n* (%)**	***n* (%)**
Currently breastfeeding	No	85(39.0)	156(35.8)
	Yes	133(61.0)	280(64.2)
Supplementary food	< 6 month	9(4.1)	15(3.4)
	At 6 months	182(83.5)	359(82.3)
	> 6 month	27(12.4)	62(14.2)
Rotavirus vaccination	No	1(0.5)	9(2.1)
	Yes	217(99.5)	427(97.9)
Measles vaccination	No	23(10.6)	53(12.2)
	Yes	195(89.4)	383(87.8)
Vitamin-A supplementation	No	12(5.5)	23(5.3)
	Yes	206(94.5)	413(94.7)
Type of water collection container	Pot	3(1.4)	8(1.8)
	Plastic bucket	0(0.0)	4(0.9)
	Jerry can	215(98.6)	424(97.2)
Storage container washed before fetching water	No	38(17.4)	123(28.2)
	Yes	180(82.6)	313(71.8)
Frequency of washing of water storage container per week	1–4	115(52.8)	275(63.1)
	> 4	65(29.8)	38(8.7)
Water drawing method	Pouring	109(50.0)	194(44.5)
	Dipping	68(31.2)	235(53.9)
	Both	41(18.8)	7(1.6)
Water treatment at home	No	125(57.3)	303(69.5)
	Yes	93(42.7)	133(30.5)
How often do you treat water?	Sometimes	59(27.1)	114(26.4)
	Daily	41(18.8)	20(4.6)
Method of water treatment	Strain through cloth	33(15.1)	54(12.4)
	Boil	63(28.9)	47(10.8)
	Chlorine	4(1.8)	33(7.6)
Wash hands at critical times (per day)	1–2	105(48.2)	194(44.5)
	3–5	113(51.8)	242(55.5)
Material used for handwashing	Water only	120(55.0)	199(45.6)
	Water & soap	98(45.0)	237(54.4)
Feces seen around pit hole	Yes	33(15.1)	157(36.0)
	No	160(73.4)	152(34.9)
Feces seen around the compound	Yes	34(15.6)	169(38.8)
	No	184(84.4)	267(61.2)
Mother/caregiver history of diarrhea within last two weeks	Yes	9(4.1)	11(2.5)
	No	209(95.9)	425(97.5)
Child history of acute diarrhea within last two weeks	No	195(89.4)	333(76.4)
	Yes	23(10.6)	80(18.3)

### Prevalence of acute diarrhea

The overall two-week acute diarrhea prevalence in the study was 15.7% (95%, CI: 13.1–18.7). The prevalence of acute diarrhea during the two weeks prior to the survey among children under five living in CLTSH-implementing *kebeles* was 10.6% (95% CI: 6.6–14.7) and in non-CLTSH-implementing *kebeles* 18.3% (95%, CI:14.8–22.2).

### Factors associated with acute diarrhea in CLTSH-implementing *kebeles*

In this study, some variables were found in the bi-variable analysis to be significantly associated with acute diarrhea; those with *p*-values < 0.25 were analyzed in the multivariable analysis to determine the related effects of the independent variables on the occurrence of acute diarrhea (Table 5).

**Table 5:** Bi-variable logistic regression analysis of association of variables with under-five acute diarrhea among CLTSH-implementing, non-implementing *kebeles* and pooled estimate in Menz Gera Midir District, North Shoa Zone, and Amhara Region, Ethiopia, February and March 2020

**Variable**	**Category**	**Model 1: CLTSH-implementing**				**Model 2: Non-CLTHS-implementing**				**Model 3 (Pooled Analysis)**			
		**Acute diarrhea**		**COR**	***P*-value**	**Acute diarrhea**		**COR**	***P*-value**	**Acute diarrhea**		**COR**	***P*-value**
		No	Yes			No	Yes			No	Yes		
Relation to child	Caregiver	31	6	1.86(0.68–5.11)	0.220	88	24	1.30(0.76–2.22)	0.330	119	30	1.49(0.93–2.38)	0.091
	Mother	164	17	Ref		268	56	Ref		432	73	Ref	
Mother’s education	Illiterate	31	5	1.69(0.47–5.98)	0.410	55	16	1.00(0.48–2.08)	0.981	86	21	1.21(0.64–2.26)	0.541
	Read and write	7	2	3.00(0.50–17.80	0.221	14	1	0.24(0.03–1.98)	0.182	21	3	0.70(0.19–2.54)	0.593
	Primary school	94	10	1.11(0.38–3.22)	0.831	211	41	0.67(0.37–1.20)	0.173	305	51	0.83(0.50–1.37)	0.464
	Secondary or above	63	6	Ref		76	22	Ref		139	28	Ref	
Household size (persons)	> 5	61	10	1.69(0.70–4.06)	0.243	127	24	0.77(0.45–1.30)	0.336	188	34	0.95(0.60–1.48)	0.828
	1–5	134	13	Ref		229	56	Ref		363	69	Ref	
Sex of child	Female	110	10	0.59(0.24–1.42)	0.244	181	33	0.67(0.41–1.10)	0.121	291	43	0.64(0.41–0.98)	0.043
	Male	85	13	Ref		175	47	Ref		260	60	Ref	
Age of child (months)	6–11	20	3	2.35(0.43–12.65)	0.325	39	9	0.94(0.39-.28)	0.901	59	12	1.15(0.53–2.49)	0.710
	12–23	37	7	2.96(0.71–12.25)	0.136	76	16	0.86(0.41–1.80)	0.692	113	23	1,15(0.61–2.18)	0.650
	24–35	47	4	1.33(0.28–6.28)	0.717	93	21	0.92(0.46–1.84)	0.823	140	25	1.01(0.54–1.88)	0.964
	36–47	44	6	2.13(0.50–9.06)	0.308	70	15	0.88(0.41–1.86)	0.738	114	21	1.04(0.54–2.00)	0.891
	48–59	47	3	Ref		78	19	Ref		125	22	Ref	
Number of under-five children per household	Two or more	18	4	2.07(0.63–6.75)	0.229	56	17	1.44(0.78–2.65)	0.231	74	21	1.65(0.96–2.82)	0.061
	One	177	19	Ref		300	63	Ref		477	82	Ref	
Monthly household income ($, USD)	< 15.0	152	17	0.47(0.14–1.57)	0.221	285	56	0.56(0.22–1.39)	0.213	437	73	0.56(0.27–1.15)	0.111
	15.0–22.7	26	2	0.32(0.05–1.98)	0.224	51	17	0.95(0.34–2.64)	0.924	77	19	0.83(0.35–192)	0.661
	> 22.7	17	4	Ref		20	7	Ref		37	11	Ref	
Source of drinking water	Unimproved	60	10	1.73(0.71–4.16)	0.223	96	45	**3.48(2.11–5.74)**	< 0.001	156	55	2.90(1.88–4.45)	< 0.001
	Improved	135	13	Ref		260	35	Ref		395	48	Ref	
Walking distance to water source (minutes)	> 30	128	20	3.49(1.00–12.16)	0.054	279	58	0.72(0.41–1.26)	0.251	407	78	1.10(0.67–1.80)	0.690
	≤ 30	67	3	Ref		77	22	Ref		144	25	Ref	
Latrine availability	No	26	1	0.29(0.03–2.28)	0.245	97	33	**1.87(1.13–3.09)**	0.011	123	34	1.71(1.08–2.70)	0.028
	Yes	169	22	Ref		259	47	Ref		428	69	Ref	
Average daily water consumption per person (liters)	< 20	153	19	1.30(0.42–4.04)	0.641	293	61	0.69(0.38–1.23)	0.212	446	80	0.81(0.49–1.36)	0.445
	≥ 20	42	4	Ref		63	19	Ref		105	23	Ref	
Child feces disposal	Outside latrine	50	10	2.23(0.92–5.40)	0.072	202	62	**2.62(1.49–4.62)**	< 0.001	252	72	2.75(1.75–4.33)	< 0.001
	Inside latrine	145	13	Ref		154	18	Ref		299	31	Ref	
Solid waste disposal	Improper	46	10	**2.49(1.02–6.05)**	0.043	144	51	**2.58(1.56–4.28)**	0.001	190	61	2.76(1.79–4.24)	0.001
	Proper	149	13	Ref		212	29	Ref		361	42	Ref	
Liquid waste disposal	Improper	68	11	1.71(0.71–4.08)	0.227	120	40	**1.78(1.09–2.90)**	0.021	196	51	1.77(1.16–2.71)	0.008
	Proper	127	12	Ref		228	40	Ref		355	52	Ref	
Livestock kept in house	Yes	45	8	1.77(0.70–4.46)	0.225	71	15	0.92(0.49–1.72)	0.801	116	23	1.07(0.64–1.79)	0.770
	No	150	15	Ref		285	65	Ref		435	80	Ref	
Currently breastfeeding	No	79	6	0.51(0.19–1.37)	0.184	132	24	0.72(0.43–1.22)	0.231	211	30	0.66(0.41–1.04)	0.071
	Yes	116	17	Ref		224	56	Ref		340	73	Ref	
Home water treatment	No	107	18	**2.96(1.05–8.29)**	0.038	237	66	**2.36(1.27–4.38)**	0.001	344	84	2.66(1.57–4.50)	< 0.001
	Yes	88	5	Ref		119	14	Ref		207	19	Ref	
Water drawing method	Pouring	96	13	Ref	0.849	163	31	Ref		259	44	Ref	
	Dipping	61	7	0.84(0.32–2.24)	0.588	189	46	1.28(0.77–2.11)	0.330	250	53	1.24(0.80–1.93)	0.310
	Both	38	3	0.58(0.15–2.16)	0.083	4	3	3.94(0.84–18.49)	0.081	42	6	0.84(0.33–2.09)	0.71
Handwashing at critical times per day	1–2	89	16	**2.72(1.07–6.91)**	0.034	142	52	**2.79(1.68–4.64)**	0 < .001	231	68	2.69(1.73–4.18)	< 0.001
	3–5	106	7	Ref		214	28	Ref		320	35	Ref	
Handwashing material	Water only	102	18	**3.28(1.17–9.19)**	0.025	157	42	1.40(0.86–2.27)	0.174	259	60	1.57(1.02–2.40)	0.031
	Water and soap/ash	93	5	Ref		199	38	Ref		292	43	Ref	
Feces seen in compound	Yes	28	6	2.10(0.76–5.79)	0.151	133	36	1.37(0.84–2.23)	0.201	161	42	1.66(1.08–2.57)	0.029
	No	167	17	Ref		223	44	Ref		390	61	Ref	
CLTSH status of *kebele*	Implemented									195	23	0.32(0.23–0.86)	< 0.001
	Not implemented									356	80	Ref	

Ref, reference category

We found that implementing the CLTSH program was a protective factor for acute diarrhea (AOR: 0.24; 95% CI: 0.20–0.6). In the *kebeles* where CLTSH had been implemented, the odds of developing acute diarrhea among children of mothers/caregivers who did not wash their hands at critical times were 3.02 times (AOR: 3.02; 95% CI: 1.12–8.12) higher than those who did wash their hands at critical times. Children in those *kebeles* whose mothers/caregivers used only water to wash their hands were 3.28 times (AOR: 3.28; 95% CI: 1.13–9.56) more likely to develop acute diarrhea than children whose mothers/caregivers used water with soap or other detergent material to wash their hands (Table 6).

**Table 6:** Multivariable regression analysis of association of variables with under-five acute diarrhea among CLTSH-implementing, non-implementing *kebeles* and pooled estimate in Menz Gera Midir District, North Shoa Zone Amhara Region, Ethiopia, February and March 2020

Variable	Category	Model 1: CLTSH-implemented					Model 2: Non-CLTHS-implemented				Model 3 (Pooled analysis)			
		**Acute diarrhea**			**AOR**	***P*-value**	**Acute diarrhea**		**AOR**	***P*-value**	**Acute diarrhea**		AOR	***P*-value**
		**No**	**Yes**				**No**	**Yes**			**No**	**Yes**		
Relation to child	Caregiver	31	6		2.28(0.64–8.09)	0.200	88	24	1.47(0.81–2.68)	0.200	119	30	1.42(0.84–2.41)	0.180
	Mother	164	17		Ref		268	56	Ref		432	73	Ref	
Mother’s education	Illiterate	31	5		1.65(0.30–9.00)	0.560	55	16	0.87(0.38–1.98)	0.740	86	21	-	
	Read & write	7	2		2.29(0.21–24.01)	0.480	14	1	0.22(0.02–1.94)	0.170	21	3	-	
	Primary school	94	10		0.83(0.22–3.09)	0.790	211	41	0.60(0.31–1.15)	0.130	305	51	-	
	Secondary or above	63	6		Ref		76	22	Ref		139	28	-	
Household size (persons)	> 5	61	10		1.78(0.69–4.63)	0.230	127	24	0.51(0.27–0.94)		188	34	-	
	1–5	134	13		Ref		229	56	Ref		363	69	-	
Sex of child	Female	110	10		1.12(0.33–3.75)	0.850	181	33	0.67(0.39–1.14)	0.140	291	43	0.64(0.40–1.01)	0.060
	Male	85	13		Ref		175	47	Ref		260	60	Ref	
Age of child (months)	6–11	20	3		5.32(0.69–40.93)	0.100	39	9	-		59	12	-	
	12–23	37	7		2.38(0.44–12.87)	0.310	76	16	-		113	23	-	
	24–35	47	4		1.31(0.22–7.60)	0.750	93	21	-		140	25	-	
	36–47	44	6		4.84(0.85–27.46)	0.070	70	15	-		114	21	-	
	48–59	47	3		Ref		78	19	-		125	22		
Number of under-five children per household	Two or more	18	4		2.23(0.44–11.22)	0.320	56	17	1.65(0.83–3.24)	0.140	74	21	1.69(0.93–3.08)	0.080
	One	177	19		Ref		300	63	Ref		477	82	Ref	
Monthly household income ($, USD)	< 15	152	17		0.39(0.09–1.74)	0.220	285	56	0.53(0.18–1.52)	0.230	437	73	0.46(0.20–1.04)	0.060
	15–22.7	26	2		0.21(0.02–1.92)	0.160	51	17	0.95(0.29–3.09)	0.930	77	19	0.60(0.23–1.56)	0.300
	> 22.7	17	4		1		20	7	1		37	11	1	
Source of drinking water	Unimproved	60	10		1.98(0.75–5.22)	0.160	96	45	**2.81(1.65–4.78)**	< 0.001	156	55	**2.56(1.62–4.05)**	< 0.001
	Improved	135	13				260	35	Ref		395	48	Ref	
Walking distance to water source (minutes)	> 30	128	20		3.31(0.91–12.07)	0.060	279	58	-		407	78	-	
	≤ 30	67	3		Ref		77	22	-		144	25	-	
Latrine availability	No	26	1		0.26(0.03–2.28)	0.220	97	33	1.66(0.96–2.89)	0.069	123	34	1.71(0.43–1.20)	0.200
	Yes	169	22		Ref		259	47	Ref		428	69	Ref	
Average daily water consumption per person (liters)	< 20	153	19		-	-	293	61	0.84(0.43–1.63))	0.610	446	80	-	
	≥ 20	42	4		-	-	63	19	1		105	23	-	
Child feces disposal	Outside latrine	50	10		1.85(0.64–5.27)	0.240	202	62	**2.10(1.13–3.89)**	0.010	252	72	**2.20(1.34–3.60)**	< 0.001
	Inside latrine	145	13		Ref		154	18			299	31	Ref	
Solid waste disposal	Improper	46	10		2.35(0.91–6.04)	0.070	144	51	**1.95(1.12–3.38)**	0.010	190	61	**2.19(1.36–3.53)**	< 0.001
	Proper	149	13		Ref		212	29	Ref		361	42	Ref	
Liquid waste disposal	Improper	68	11		0.95(0.30–3.05)	0.940	120	40	1.13(0.60–2.12)	0.690	196	51	1.06(0.64–1.76)	0.810
	Proper	127	12		Ref		228	40	Ref		355	52	Ref	
Livestock kept in house	Yes	45	8		1.61(0.52–4.96)	0.400	71	15	-		116	23	-	
	No	150	15		Ref		285	65	-		435	80	-	
Currently breastfeeding	No	79	6		0.87(0.16–4.70)	0.870	132	24	0.78(0.43–1.42)	0.420	211	30	0.68(0.41–1.14)	0.14
	Yes	116	17		Ref		224	56	Ref		340	73	Ref	
Home water treatment	No	107	18		2.77(0.94–8.13)	0.060	237	66	**2.33(1.21–4.49)**	0.010	344	84	**2.53(1.45–4.40)**	0.001
	Yes	88	5		Ref	-	119	14	Ref		207	19	Ref	
Water drawing method	Pouring	96	13		**-**	-	163	31	Ref	0.540	259	44	-	
	Dipping	61	7		-	-	189	46	1.19(0.67–2.10)	0.750	250	53	-	
	Both	38	3		**-**	-	4	3	4.29(0.75–24.40)		42	6		
Handwashing at critical times per day	1–2	89	16		**3.02(1.12–8.12)**	0.020	142	52	**2.57(1.49–4.42)**	0.001	231	68	**2.54(1.59–4.06)**	< 0.001
	3–5	106	7		Ref		214	28	Ref		320	35	Ref	
Hand washing material	Only water	102	18		**3.28(1.13–9.58)**	0.020	157	42	1.20(0.68–2.10)	0.520	259	60	1.28(0.80–2.07)	0.290
	Water and soap/ash	93	5		Ref		199	38	Ref		292	43	Ref	
Feces seen in compound	Yes	28	6		2.35(0.75–7.33)	0.140	133	36	0.86(0.48–1.56)	0.630	161	42	0.95(0.56–1.60)	0.860
	No	167	17		Ref		223	44	Ref		390	61	Ref	
CLTSH status of *kebele*	Implemented										195	23	0.24(0.20–0.6)	< 0.001
	Non-implemented										356	80	Ref	

Ref, reference category

### Factors associated with acute diarrhea in non-CLTSH-implementing *kebeles*

In *kebeles* where CLTSH had not been implemented, the odds of acute diarrhea were 2.81 times (AOR: 2.81; 95% CI: 1.65–4.78) higher among children of mothers/caregivers who fetched water from an unimproved drinking water source than among those whose mothers/caregivers fetched water from an improved water source. In the non-implementing *kebeles*, the odds of developing acute diarrhea among under-five children whose mothers/caregivers practiced unsafe disposal of child feces were 2.1 times (AOR: 2.10; 95% CI:1.13–3.89) higher than among those children whose mothers/caregivers practiced safe disposal of child feces (Table 6).

In the non-implementing *kebeles*, children whose mothers/caregivers disposed of solid waste improperly were 1.95 times (AOR: 1.95; 95% CI: 1.12–3.38) more likely to develop acute diarrhea than children whose mothers/caregivers disposed of solid waste properly. The occurrence of acute diarrhea was 2.33 times (AOR: 2.33; 95% CI: 1.21–4.49) higher among children whose households did not treat drinking water compared to children whose households did treat drinking water. In non-implementing *kebeles*, the odds of developing acute diarrhea were 2.57 times (AOR: 2.57; 95% CI: 1.49–4.42) higher among children whose mothers/caregivers didn’t wash their hands at critical times than among those whose mothers/caregivers did wash their hands at critical times (Table 6).

### Factors associated with acute diarrhea from pooled multivariable analysis

In the pooled multivariable analysis, the odds of acute diarrhea were 2.5 times (AOR: 2.56; 95% CI: 1.62–4.05) higher among children of mothers/caregivers who fetched water from an unimproved water source compared to children of mothers/caregivers who fetched water from an improved water source. This analysis also found that the odds of developing acute diarrhea among under-five children whose mothers/caregivers practiced unsafe disposal of child feces were 2.2 times (AOR: 2.20; 95% CI: 1.34–3.60) higher than children those children whose mothers/caregivers practiced safe disposal of child feces. Children whose mothers/caregivers disposed solid waste improperly were 2.19 times (AOR: 2.19; 95% CI: 1.36–3.53) more likely to develop acute diarrhea than children whose mother/caregivers disposed of solid waste properly (Table 6).

Pooled multivariable analysis also revealed that the odds of acute diarrhea were 2.53 times higher (AOR: 2.53; 95% CI: 1.45–4.40) among children in households that did not treat their drinking water than those in households that did treat it. Children whose mothers/caregivers did not wash their hands daily at critical times were 2.54 times (AOR: 2.54; 95% CI: 1.59–4.06) more likely to develop acute diarrhea than children whose mother/caregivers washed their hands at critical times. Implementation of the CLTSH program was also a preventive factor (AOR: 0.24; 95%: 0.20–0.60) for acute diarrhea compared to not implementing CLTSH (Table 6).

## Discussion

We conducted a comparative cross-sectional study in CLTSH-implementing and non-implementing *kebeles* to investigate the prevalence of diarrhea and associated factors among children under five. We found the prevalence of acute diarrhea among children under five living in CLTSH-implementing *kebeles* to be 10.6% (95% CI: 6.6–14.7) and among those that had not implemented CLTSH 18.3% (95%CI:14.8–22.2).

The prevalence of acute diarrhea among CLTSH-implementing areas in Menz Gera Midir District similar to reports from Kenya (11.1%) [26] and rural Dangla District, Ethiopia (9.9%) [14]. However, this rate is lower than rates reported from rural Mali (22.0%) [27], Kersa District in Ethiopia (18.9%) [15], and Yaya Gulele District in Ethiopia (13.4%) [28]. The lower rate in our study might be due to effective monitoring, follow-up, and prohibition and declaration of open defecation-free *kebeles* after the CLTSH intervention.

In CLTSH-implementing *kebeles*, children whose households used only water for washing hands were 3.0 times more likely to develop acute diarrhea than children whose households used water and soap or other detergents for washing hands. This result is supported by other Ethiopian studies [21, 29]. Similarly, in this group the occurrence of acute diarrhea was higher among children whose mothers/caregivers did not wash their hands at critical times than among children whose mothers/caregivers washed their hands at critical times. This result agrees with studies in other Ethiopian communities [21, 30–34]. This pattern might be due to inadequate hand hygiene promotion in both CLTSH-implementing and non-implementing *kebeles.*

The prevalence of two-week acute diarrhea morbidity among children under five living in non-CLTSH-implementing *kebeles* in our study was 18.3% (95% CI: 14.8–22.2). This is lower than found in studies in Kenya (21.6%), Mali (24%), Yaya Gulele in Ethiopia (36.3%), and Kersa, Ethiopia (22.2%), areas that also lack implementation of CLTSH [15, 27, 28, 35]. These variations in prevalence might be due to differences in the performance and implementation of CLTSH packages across countries.

This study shows the prevalence of acute diarrhea in households in non-CLTSH-implementing *kebeles* to be significantly higher than in households in CLTSH-implementing *kebeles*. The higher rate might be due to effective implementation of the CLTSH strategy, a higher level of awareness about WASH and committed administrators in implementing *kebeles*, variations in coverage and utilization of the health extension package, and effective social mobilization programs in Gera Midir District.

Improperly disposed child feces are accessed by flies that then contaminate food and water by pathogenic organisms. In this study, unsafe child feces disposal was independently associated with diarrhea. Children whose households did not dispose of child feces safely in latrines were 2.0 times more likely to develop diarrhea than children whose parents properly disposed feces. In rural Bangladesh and Benishangul Gumuz Region in Ethiopia, unsafe disposal of children’s feces was significantly associated with the occurrence of diarrhea [28, 36, 37]. Reasons for these variations may be differences in educational level of the communities and inadequate follow-up and monitoring activities.

The finding that unimproved drinking water sources were significantly associated with acute diarrhea disease in non-CLTSH-implementing *kebeles* corroborates results of other studies [31, 32]. In this study, the occurrence of acute diarrhea was 2.81 times higher among households using water from unimproved sources compared to households using improved water sources. This might be due to the *kebeles’* accessibility to water sources and unaffordability of installing improved drinking water supplies.

The overall prevalence of acute diarrhea in Menz Gera Midir District (pooled analysis) was 15.7 (95% CI: 13.1–18.7), which is much higher than reported by studies in slums of Addis Ababa (11.9%) [38] and in Dale District in southern Ethiopia (13.6%) [39]. However, the prevalence of under-five acute diarrhea in this study was lower than in cross-sectional studies in other parts of Ethiopia, including Arba Minch District (30.5%) [40], North Gondar Zone (22.1%) [41], Dejen District (23.8%) [42], and Hadaleala District (26.1%) [43]. But our results are similar with to other Ethiopian community-based cross-sectional studies in Bahr Dar City (14.5%) [30], Kamashi District (14.5%) [44], and Debre Berhan Town (16.4%) [45]. These differences might be due to variations in the age and sex distribution of samples, geographical location, and socioeconomic status of the population.

From the pooled analysis, use of unimproved drinking water sources was 2.5 times more likely to be associated with acute diarrhea than use of improved sources. This result agrees with some studies in Ethiopia [6, 29, 42]. The possible explanation for these finding might be lack of improved water source availability, poor performance of home-based water treatment, and low latrine coverage.

Children whose mothers or caregivers practiced unsafe child feces disposal were 2.0 times more vulnerable to acute diarrhea than children whose mothers or caregivers safely disposed of child feces in latrines. This result corroborates studies in Ethiopia and rural Bangladesh [34, 37, 42, 46]. This pattern may be due to pathogens in feces being disposed outside of latrines and children coming in contact with feces during playing. Similarly, the risk of diarrhea was 2.16 times greater in households that did not dispose of solid waste properly compared to households that did. This finding agrees with studies conducted in Dale District, southern Ethiopia [47]. This might be due to improper solid waste disposal, which exacerbates breeding of insect vectors of diarrheal pathogens.

The odds of developing acute diarrhea were 2.5 times higher among children whose mothers/caregivers did not wash their hands at critical times than among children whose mothers/caregivers practiced hand washing at critical times. This result agrees with studies in Arba Minch District [40], and Kamashi District in western Ethiopia [44] and might be due to the fact that human hands are primary vehicles for transmitting diarrheal infections. Children in households that did not treat their drinking water were 2.56 times more likely to develop acute diarrhea than children in households that used a water treatment method, a finding similar to those of other studies in Ethiopia [46, 48, 49].

### Limitations of the study

The limitations of this study included the fact that it was not a randomized controlled trial, the unknown content and quality of CLTSH implementation, the self-reporting, not observing of many of the behavioral factors, the diarrhea being self-reported, that the study did not investigate the impact of seasonal variation on the occurrence of acute diarrhea, shortening the multiple comparisons with other studies and recall bias of the study participant.

## Conclusion

Our findings show that the prevalence of acute diarrhea in CLTSH-implementing *kebeles* was lower than in non-CLTSH-implementing *kebeles* in Menz Gera Midir District. We also found that implementing the CLTSH program was a protective factor for acute diarrhea. In non-CLTSH-implementing *kebeles*, unimproved water sources, unsafe disposal of child feces outside of latrines, improper solid waste disposal, untreated drinking water, and failure to wash hands at critical times were important factors in the occurrence of diarrhea. These findings suggest that CLTSH implementation can have a positive impact on acute diarrhea prevention. Therefore, strengthening CLTSH programs and expanding them to other areas are highly recommended.

## Supplementary Information

**Additional file 1.** 

## Data Availability

Data and all the materials will be available from the corresponding author upon request.

## Abbreviations

AOR: Adjusted odds ratio;
COR: Crude odds ratio;
CLTSH: Community-Led Total Sanitation and Hygiene;
WASH: Water, Sanitation, and Hygiene
