# Supplementary material for: Prevalence of and factors associated with acute diarrhea among children under five in rural areas in Ethiopia with and without implementation of community-led total sanitation and hygiene
Source: BMC Pediatr. 2022 Mar 21;22:148. doi: 10.1186/s12887-022-03202-8 (PMC8935707; doi:10.1186/s12887-022-03202-8)
Supplement: Supplementary file 1 — Additional file 1. [file 12887_2022_3202_MOESM1_ESM.docx]

**Tick (✓) in the box**

Non-CLTSH implemented

CLTSH implemented

**Identification**

Questionnaire ID---------------------------------------

Date of the interview (-------/----------/---------------

District----------------------- Kebele --------------------Gott………………….. House number: -----------------------------------

Name of Interviewer---------------------------------------- and signature----------------------------------

Completed/uncompleted questionnaires (tick (✓) it after the end of the interview)

Completed Uncompleted

| **Part I: SOCIO-DEMOGRAPHIC FACTORS** | | | | | |
| --- | --- | --- | --- | --- | --- |
| **S.No** | **Questions** | **Responses** | | | **Skip pattern** |
| 101 | Relation of the respondent to the child | 1. Mother 2. Caregiver | | |  |
| 102 | Age of the mother/caregiver (years)? | ……..year | | |  |
| 103 | Sex of the mother/caregiver? | 1. Male 2. female | | |  |
| 104 | Marital status of the mother/caregiver? | 1. Married  2. Single  3. Windowed  4. Divorced | | |  |
| 105 | Mother/caregiver educational status? | ………………….. | | |  |
| 106 | If the answer for Q, no 104 is married what is the educational status of the father? | ………………………………. | | |  |
| 107 | Religion of primary caregiver? | 1. Orthodox Tewahido 2. Muslim  3. Protestant 4. Catholic  5. others | | |  |
| 108 | Occupation of the primary caregiver? | 1. Housewife  2. Civil servant  3. Merchant  4. Other/specify………………….. | | |  |
| 109 | Number of household members? | -------------------------------- | | |  |
|  | **Children under-five years old related factors** |  | | |  |
| 110 | Sex of the child | 1. Male 2. Female | | |  |
| 111 | Age of the child/ month/ | ……..month | | |  |
| 112 | A total number of under-five children? | ………………. | | |  |
| 113 | Birth order of the child | …………….. | | |  |
| 114 | Average monthly incomes of the family | …………. | | |  |
|  | PART II. ENVIRONMENTAL HEALTH CONDITIONS | | | |  |
| 201 | What is the source of your drinking water? | 1. Public tap  2. Spring  3. Hand dug well  4. River  5. Others/Specify…… | | |  |
| 202 | How much time it takes to reach on your drinking water source? | ---- --------------minutes | | |  |
| 203 | How many liters of water do you consume per day per person? /Calculated by the researcher/ | ----------20 liter jurican | | |  |
| 204 | Does is there is water source interruption? | 1. Yes 2. No | | |  |
| 205 | Does latrine available? | 1. Yes 2. No | | | If no, skip to no 215 |
| 206 | If the answer for Q, no 205 is yes what is the owner of the latrine? | 1. Share 2. Privately owned | | |  |
| 207 | If the answer for Q, no 205 is yes What type of latrine? | 1. Traditional pit latrine 2. Improved pit latrine   3. Other specify…….. | | |  |
| 208 | If the answer for Q, no 205 is yes Does latrine seat hole cover during the survey **( observe)** | 1. Yes 2. No | | |  |
| 209 | If the answer for Q, no 206 is shared, how many households utilize? | ……….. | | | If privet, skip to no 210 |
| 210 | Where did you dispose child faeces that do not start to use latrine? | 1. Inside the compound  2. Outside the compound  3. In the latrine  4. Other, specify------------- | | |  |
| 211 | How often do you clean the latrine? | 1. Daily 2. Sometimes 3. Never | | |  |
| 212 | The average distance of the latrine from the house | ……………………. | | |  |
| 213 | Do you have a handwashing facility near the toilet? **(observe)** | 1. Yes 2. No | | |  |
| 214 | If ‘no’ for Q no 206, where does the family defecates? | 1. Open field 2. Other/Specify. | | |  |
| 215 | Where do you dispose of refuse/garbage? | 1. Pit 2. Open field 3. Stored outside the compound   4. Other specify…………. | | |  |
| 216 | How you dispose of the household’s wastewater? | 1. Sock pit 2. Spray open field   3. Other specify………… | | |  |
| 217 | Do you always clean your house? | 1. Yes 2. No | | |  |
| 218 | Do you keep domestic animals with you in the same house? | 1. Yes 2. No | | |  |
|  | PART III. BEHAVIORAL ASPECTS |  | | |  |
| 301 | Have you ever breastfed your child? | 1. Yes 2. No | | |  |
| 302 | For how long did you breastfeed your child? | …………month | | |  |
| 303 | What is his/her current breastfeeding status? | 1. Yes 2. No | | |  |
| 304 | At what age the child started supplementary food? | ………………….month | | |  |
| 305 | Does prepared special meal for child? | 1. Yes 2. No | | |  |
| 306 | How many times does he/she eat per day? | ……………. | | |  |
| 307 | Did the child receive Rota vaccination | 1. Yes 2. No | | |  |
| 308 | Did the child receive measles vaccination? | 1. Yes 2. No | | |  |
| 309 | Did the child receive Vit A supplementation | 1. Yes 2. No | | |  |
| 310 | What type of water collection container during the last two weeks? | 1. Pot 2. Plastic bucket 3. Iron bucket 4. Jerry can 5. Other specify…………. | | |  |
| 311 | How did you transport the collected drinking water to the house during the last two weeks? | 1. In a covered container 2. In an uncovered container 3. Covered by leaves 4. Other (specify)…… | | |  |
| 312 | Did you wash water storage before fetching? | 1. Yes 2. No | | |  |
| 313 | Frequency of cleaning water storage containers during the last two weeks? | ………….. | | |  |
| 314 | Does the drinking-water storage container have a cover? **Observe** | 1. Yes 2. No | | |  |
| 315 | How do you take water from the drinking water storage container? | 1. Pouring 2. Dipping 3. Both | | |  |
| 316 | Do you treat your water at the household | 1. Yes 2. No | | |  |
| 317 | If the answer for Q, no 316 is yes, how often do you treat the water? | 1. Daily  2. Sometimes  3. Other specify………. | | |  |
| 318 | What method do you use to treat drinking water? | 1. Boiling 2. Chlorine 3. Strain through a cloth   4. Others specify………. | | |  |
| 319 | Do you wash your hands? | 1. Yes 2. No | | |  |
| 320 | If the answer for Q, no 316 is yes, when do you wash your hands? (More than one)? | 1. Before food Preparing | Yes | No |  |
|  |  | 2. Before the child Feeding | Yes | No |  |
|  |  | 3. After the toilet visit | Yes | No |  |
|  |  | 4. After cleaning the child anus | Yes | No |  |
|  |  | 5. Before eating | Yes | No |  |
| 321 | Cleansing material for handwashing? | 1. Water only  2. Soap or ash  3. Others specify….…….. | | |  |
| 322 | Is feces seen around the pit-hole (or on the floor)? (**observe**) | 1. Yes 2. No | | |  |
| 323 | Do feces see around the compound? (**Observe**) | 1. Yes 2. No | | |  |
| 324 | Do you (the mother/caregiver) have a history of diarrhea in the past two weeks? | 1. Yes 2. No | | |  |
| 325 | Does your child have diarrhea last two weeks? | 1. Yes 2. No | | |  |

Date of interview-------------------------Name of the interviewer----------------------------- Signature-----------------------
